# Preliminary Investigation of the Mechanical Anisotropy of the Normal Human Corneal Stroma

**DOI:** 10.1155/2018/5392041

**Published:** 2018-10-17

**Authors:** Chao Xue, Yaoqi Xiang, Min Shen, Di Wu, Yan Wang

**Affiliations:** ^1^Clinical College of Ophthalmology, Tianjin Medical University, Tianjin Eye Hospital, Tianjin Key Laboratory of Ophthalmology and Visual Science, Tianjin Eye Institute, Tianjin, China; ^2^School of Mechanical Engineering, Tianjin University, Tianjin, China

## Abstract

**Purpose:**

To investigate the anisotropic characteristics of the normal human corneal stroma using fresh corneal tissue.

**Methods:**

Sixty-four corneal specimens extracted from stromal lenticules were included in this study. The specimens were cut in the temporal-nasal (horizontal) or superior-inferior (vertical) direction. Strip specimens were subjected to uniaxial tensile testing. The tensile properties of the specimens were measured and compared in the two directions.

**Results:**

The low-strain tangent modulus was statistically significantly greater in the vertical direction than in the horizontal direction (1.32 ± 0.50 MPa vs 1.17 ± 0.43 MPa; *P*=0.035), as was the high-strain tangent modulus (51.26 ± 8.23 MPa vs 43.59 ± 7.96 MPa; *P* ≤ 0.001). The elastic modulus in the vertical direction was also higher than that in horizontal direction at stresses of 0.01, 0.02, and 0.03 MPa, but not statistically significant; so, *P*=0.338, 0.373, and 0.417, respectively.

**Conclusions:**

The biomechanical behavior in normal human corneal stroma tissue is slightly stiffer in the vertical direction than in the horizontal direction. This information may aid our understanding of the biomechanical properties of the cornea and related diseases.

## 1. Introduction

The transparent cornea is a significant component of the outer ocular tunic. In addition to serving as the primary refractive component of the eye, the cornea has unique mechanical characteristics [1–3]. Mechanical anisotropy is an important property of the cornea and may provide valuable information in terms of determining the onset and severity of many diseases [4]. Meek and Boote reported that the orientation of collagen fibrils in keratoconus was different from that in a healthy cornea [5].

Previous microstructural studies found that the majority of collagen fibrils in the central region of the human cornea had a preferential orientation in the inferior-superior (vertical) or nasal-temporal (horizontal) direction [2]. Given that collagen fibrils are the main load-carrying elements of the stroma, their preferential orientation may determine the mechanical anisotropy of the cornea [6–8].

Uniaxial tensile testing has been used to compare the mechanical behavior of porcine and cadaveric human cornea strips from different directions and showed that the elastic response differs according to the cutting direction [9, 10]. However, this test cannot describe the mechanical anisotropy of the normal human cornea accurately. Fortunately, with the advent of small incision lenticule extraction (SMILE) surgery since 2011 [11], it is now possible to obtain fresh human corneal tissue, allowing direct study of mechanical anisotropy in the fresh human corneal stroma.

While the material parameters of the corneal stromal lenticules have be measured through uniaxial tensile test in the previous study [12], the purpose of this study was to investigate the mechanical anisotropy of the normal human corneal stroma using stromal lenticules extracted during SMILE as part of an effort to elucidate the biomechanical behavior of the human cornea.

## 2. Materials and Methods

### 2.1. Surgical Procedures and Preparation of Specimens

All patients underwent a systematic ophthalmic examination to confirm a healthy cornea, including but not limited to slit-lamp microscopy, corneal topography, and measurement of intraocular pressure. The study exclusion criteria were keratoconus or suspected keratoconus, active ocular or systemic disease, a clinical history of ocular surgery or trauma, and any other condition that could affect the health of the cornea.

The SMILE procedures were performed by the same experienced surgeon using the VisuMax femtosecond laser system (Carl Zeiss Meditec AG, Jena, Germany). The details of this procedure have been described elsewhere [11, 13, 14]. After creation of a refractive lenticule and a small incision, the surgeon dissected the lenticule from the surrounding tissue and extracted it through the incision.

After extraction, the 12 o'clock position of the corneal lenticule was marked with gentian violet. Each specimen was preserved in storage medium (Eusol-C; Alchima, Padova, Italy) below 4° in a refrigerator for no more than 24 hours, after which the lenticule was prepared for the experiment.

**Figure 1:**
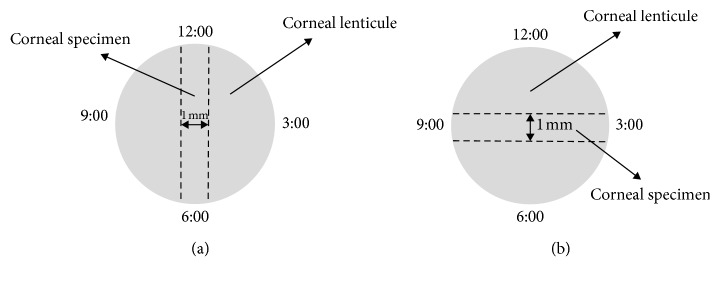
Sketch map of corneal specimen in different directions: (a) vertical; (b) horizontal.

The lenticule was gently placed on a rubber base and cut into 1.0 mm-wide strip specimens from the central region in the required direction using a customized double-blade knife. The lengths of the corneal specimens were different according to the diameter of the corneal lenticule and were approximately 6.5 mm. Specimens from left eyes were cut in the temporal-nasal (horizontal) direction, and those from right eyes were cut in the superior-inferior (vertical) direction (Figure 1).

### 2.2. Uniaxial Tensile Testing

The specimens were then clamped between the rough-surfaced jaws to prevent slippage. Next, they were subjected to uniaxial tensile testing, starting with three loading/unloading cycles to precondition the specimen, followed by loading to failure.

The tests were performed using an IBTC-50 (in situ bidirectional tension and compression) testing system (Tianjin Care Measure and Control Co., Ltd., Tianjin, China) in a laboratory water bath filled with normal saline at room temperature. The distance between the two clamps was 1.5 mm. The rate of deformation was 0.01 mm/s. The central thickness of the corneal specimen was obtained from the SMILE surgery data. The specimen width was 1 mm for this experiment.

The study was approved by the Ethics Committee at Tianjin Eye Hospital, Tianjin Medical University, and adhered to the tenets of the Declaration of Helsinki. Informed consent to use clinical data for analysis and publication was obtained from all patients.

### 2.3. Statistical Analysis

The statistical analysis was performed using IBM SPSS software version 20 (IBM Corp., Armonk, NY). Eye pairs were compared using the paired *t*-test. A *P*-value <0.05 was considered statistically significant.

## 3. Results

Sixty-four corneal specimens obtained from the left and right eyes of 32 patients (11 men; 21 women) of mean age 21.11 ± 3.24 (range 16–31) years were included in the study. The mean preoperative sphere was −4.18 ± 1.59 D in the left eyes and −4.51 ± 1.64 D in the right eyes, and there was no significant statistical significance between these two groups (*P*=0.065). Twenty‐eight of 32 left eyes and twenty‐seven of 32 right eyes have both spherical and cylindrical errors. All the astigmatism was with the rule. The mean preoperative cylinder was −0.95 ± 0.91 D in the left eyes and −0.93 ± 0.90 D in the right eyes, and there was no significant statistical significance between these two groups (*P*=0.810). The mean central lenticule thickness was 103.78 ± 25.85 *μ*m.

**Figure 2:**
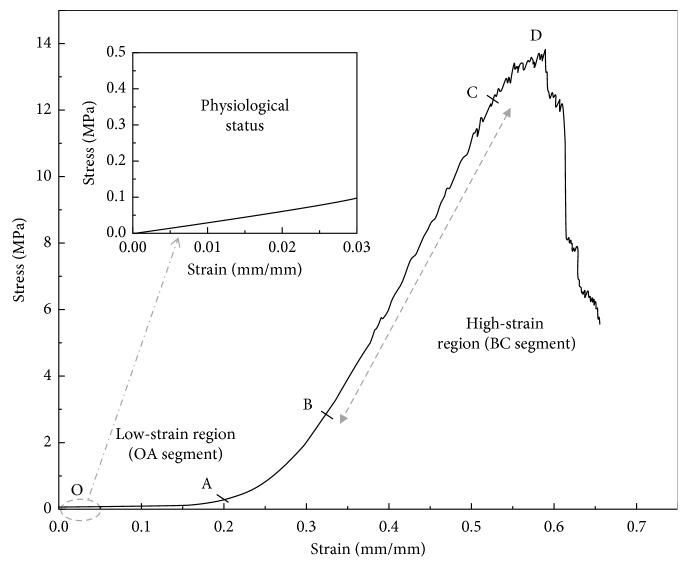
The stress-strain curve of a corneal strip.

A typical stress-strain curve is shown in Figure 2. The curve can be divided into four sections. The first section is the linear elastic OA segment, in which the load changes are very small and deformation increases rapidly. The second section is the AB segment, in which the load increases exponentially with an increase in deformation, thus reflecting a nonlinear relationship. The third section is the BC segment, which is an approximately straight line, where the maximum stress is reached at point C. The fourth section is D, which is the fracture point. The elastic modulus (*E*) of the OA segment is defined as the low-strain tangent modulus (LSTM) and that of the BC segment is defined as the high-strain tangent modulus (HSTM) [15].

**Figure 3:**
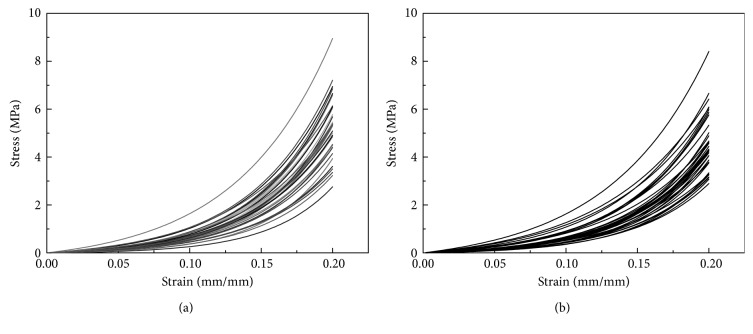
Stress-strain curves of 32 specimens in different directions: (a) vertical; (b) horizontal.

Figure 3 shows the stress-strain curves for the 64 specimens in the different directions, most of which are concentrated in a relatively small range.

**Figure 4:**
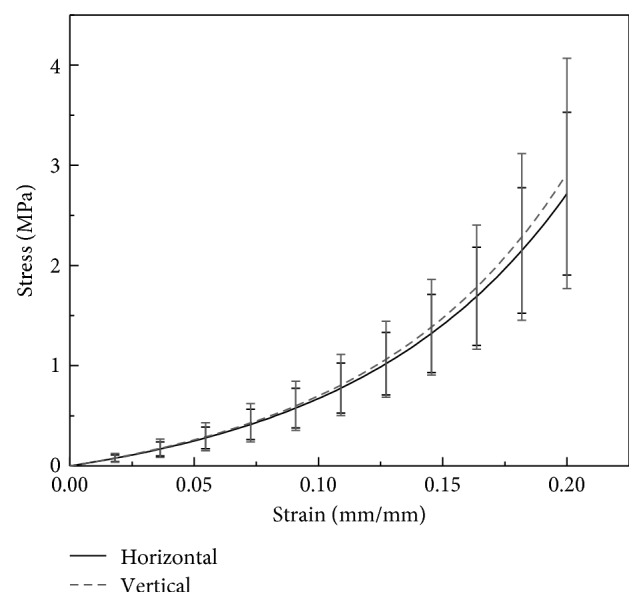
Average stress-strain curves of specimens.

Average stress-strain curves for eye pairs were compared in the vertical direction and the horizontal direction (Figure 4). The stress-strain behavior of each corneal specimen was described using the following equation:

(1) $$\begin{array}{c} \sigma =A\left(e^{B\varepsilon }-1\right), \end{array}$$

where *A* and *B* are constants [10]. The values of *A* and *B* that provided the best fit are shown in Table 1. The difference between the two curves increased with increasing strain/stress (Figure 4). Under the physiological intraocular pressure, the corneal stress is about 0.02 MPa. We calculated Young's modulus with a stress growth from 0.01 MPa to 0.03 MPa.

**Figure 5:**
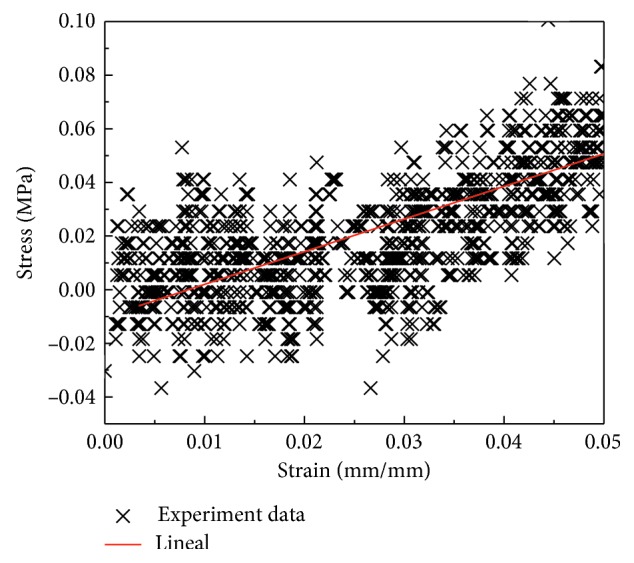
Curve fitting to obtain Young's modulus.

The uniaxial tensile test data of corneal stroma were selected. By plotting the values of *σ* for the early part of the tests (corresponding to the strain of less than 5%), we can determine Young's modulus *E* by linear regression analysis (Figure 5). On the early part of the tests, the data fluctuated greatly, and their increasing trend can be reflected by linear fitting. We used the area of strain from 0 to 0.5 as a low-strain zone, and the high-strain zones can easily be identified through the stress-strain curves. We can roughly estimate Young's modulus in low-strain zones and high-strain zones through this method.

The LSTM and HSTM values in the vertical direction were significantly higher than those in the horizontal direction (Table 2).

**Figure 6:**
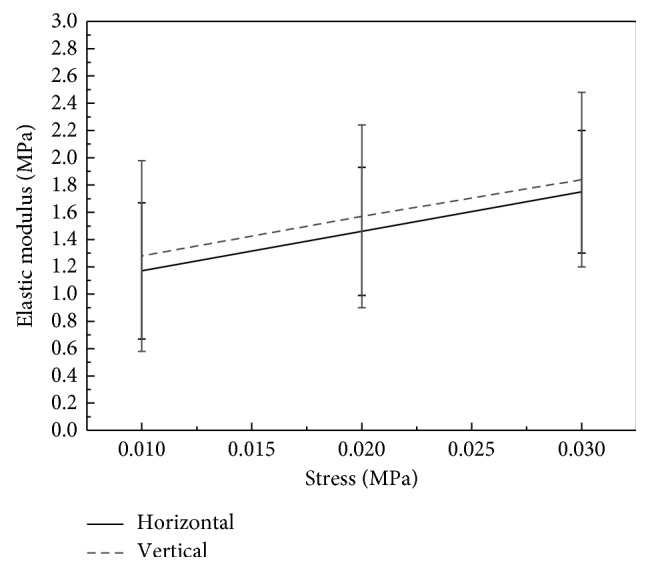
Relationships between elastic modulus and stress.

Figure 6 shows the relationship between the elastic modulus (*E*) and stress (*σ*) according to the physiologic state of the cornea. Very small scatter could be seen between the two lines, but the difference was not statistically significant (*P* > 0.05; Table 3). Figure 6 shows that the relationship between the elastic modulus and stress was linear.

## 4. Discussion

Many groups have recognized the importance of the anisotropic characteristics of the cornea, and much research effort has been focused on the biomechanical anisotropy of this structure. In previous research, the biomechanical properties of strip specimens from porcine or cadaveric human cornea tissue cut in different directions were compared using uniaxial tensile tests, and the results showed that the biomechanical behavior varied depending on the direction in which the specimen was cut [9, 10]. In one study, inflation tests were performed to investigate the global characteristics of the entire bovine cornea, and an inverse finite element method was developed to determine its anisotropic properties [16]. In another study, a computational model was used to determine the biomechanical deformation of corneal tissue that had been cut and removed and the effect of mechanical anisotropy resulting from the fibrillar architecture [17]. All these approaches provided valuable information on corneal mechanical anisotropy but could not describe the mechanical anisotropy of the normal human cornea accurately because data could only be obtained from animal or cadaveric human corneas, which may not be representative of the normal fresh human cornea. With the advent of SMILE surgery, it is now possible to obtain fresh normal corneal tissue for research purposes. Although corneal lenticules have been used in previous studies to observe changes in cell morphology [18–20], there have been no reports of biomechanical tests performed using fresh human corneal tissue.

The corneal stroma contains several hundred lamellae, each of which is composed of parallel collagen fibrils embedded in an extracellular matrix [21, 22]. The stroma comprises about 90% of the corneal thickness and determines the mechanical behavior of the cornea. Knowledge of the biomechanical properties of the corneal stroma would aid our understanding of the biomechanical properties of the cornea itself.

Eusol-C was used to preserve the corneal lenticule immediately after it was surgically extracted. The effectiveness of this storage medium in maintaining stromal hydration after 7 days of storage has been confirmed in previous studies [10, 23]. In the present study, the corneal lenticule was preserved in the storage medium, Eusol-C, for no more than 24 hours so that the specimens would maintain their good quality for the tests. Although the tensile tests were conducted in saline solution in the present study, the experiment was accomplished in a very short time; therefore, it is unlikely that significant swelling occurred during the experiments.

In this study, scatter was seen on comparison of the stress-strain curves for eye pairs in the vertical and horizontal directions. Under the same level of strain, the stress in the vertical direction was greater than that in the horizontal direction, and the difference increased with increasing strain. Both the LSTM and HSTM were significantly higher in the vertical direction than in the horizontal direction. This finding confirms that the biomechanical behavior of the cornea differs in different directions.

In this study, we analyzed the biomechanical behavior of the cornea in the physiological level of stress. Under the same level of stress, the elastic modulus of each specimen was greater in the vertical direction than in the horizontal direction at stress levels of 0.01, 0.02, and 0.03 MPa, but the difference was not statistically significant. These three stress levels were chosen because they are within the normal physiologic state of the cornea [24] and were applied immediately after the start of uniaxial tensile testing, that is, in a different phase from that in which the LSTM and HSTM were calculated. This could explain why there was a statistically significant difference in the LSTM and HSTM between the vertical direction and the horizontal direction and no statistically significant difference in the value of the elastic modulus between the two directions at stress levels of 0.01, 0.02, and 0.03 MPa. The implications of this finding require further study. In addition, there was a linear relationship between the elastic modulus and stress in this study. The cornea is a soft tissue with elastic nonlinearity properties, so the elastic modulus increases with increasing stress [9].

Previous works showed that subjects with high myopia had lower normalized corneal tangent moduli than subjects with low myopia had [25–27]. In addition, the cut pattern of the lenticule is different between pure spherical correction and those with sphere and cylinder correction; it could impact the results. In the present study, both preoperative sphere and cylinder had no significant statistical significance between eyes from horizontal and vertical directions. Based on this result, we are able to compare the tensile properties of the specimens in different directions.

Elsheikh et al. [10] reported that vertical strips of cornea from cadaveric human eyes were slightly stiffer and stronger than horizontal strips, by 10%–25% on average at a rate of deformation of 1% per minute and 16%–18% at a rate of deformation of 500% per minute. In the present study, the vertical specimens were also slightly stiffer and stronger than the horizontal specimens. We found that the vertical specimens were stronger on average than the horizontal specimens by 13% for LSTM and 18% for HSTM at a rate of deformation of 40% per minute, which is consistent with the findings of Elsheikh et al. [10]. However, in the physiological state, the difference was much smaller at stress levels of 0.01, 0.02, and 0.03 MPa, with corresponding rates of 1%, 4%, and 5%, respectively. Figures 4 and 5 show that the difference in the elastic modulus between the vertical and horizontal direction increases with increasing stress. This difference is much smaller in the very early portion of the stress-strain curve than in the later portions. The same trend was present in the study reported by Elsheikh et al. [10]. This phenomenon may also explain the much smaller difference found in the physiologic state.

In conclusion, the results of this study show that the biomechanical behavior of normal human corneal stroma tissue is slightly stiffer in the vertical direction than in the horizontal direction. To the best of our knowledge, the present study is the first attempt to investigate mechanical corneal anisotropy using fresh human corneal stroma tissue. Different elongation rates will be necessary in future studies to assess the possible effect of corneal viscoelasticity on mechanical anisotropy. Also, obtaining overall corneal properties through local property measurement will be the focus of further work.

## Figures and Tables

**Table 1:** Values of constants *A* and *B*.

No.	*A*		*B*	
	Vertical (MPa)	Horizontal (MPa)	Vertical	Horizontal
1	0.15	0.184	18.958	17.644
2	0.140	0.063	19.333	20.549
3	0.030	0.258	22.632	16.445
4	0.042	0.0720	22.771	20.021
5	0.132	0.063	18.23	21.219
6	0.075	0.075	19.502	18.987
7	0.114	0.139	19.331	17.604
8	0.222	0.127	17.548	17.683
9	0.215	0.369	17.533	14.288
10	0.276	0.238	15.732	15.774
11	0.122	0.135	19.084	17.432
12	0.119	0.119	19.516	17.446
13	0.086	0.0773	18.463	18.689
14	0.156	0.168	18.388	18.002
15	0.090	0.113	20.488	18.708
16	0.171	0.100	17.972	19.108
17	0.152	0.088	19.142	20.281
18	0.211	0.222	16.048	15.459
19	0.056	0.119	20.381	17.939
20	0.076	0.085	20.536	19.668
21	0.126	0.082	17.993	18.369
22	0.132	0.058	18.746	19.956
23	0.083	0.083	18.812	20.179
24	0.131	0.060	19.302	21.107
25	0.103	0.049	19.023	21.189
26	0.144	0.128	17.959	18.332
27	0.472	0.172	14.969	17.799
28	0.129	0.101	18.256	18.586
29	0.094	0.211	19.039	16.713
30	0.126	0.133	18.438	17.57
31	0.183	0.511	17.078	14.302
32	0.108	0.064	19.634	19.178
Mean ± SD	0.140 ± 0.08	0.140 ± 0.10	18.78 ± 1.63	18.32 ± 1.83

**Table 2:** Comparison of LSTM and HSTM in eye pairs.

Parameters	Horizontal	Vertical	*P* value
LSTM(MPa)	1.17 ± 0.43	1.32 ± 0.50	0.035
HSTM(MPa)	43.59 ± 7.96	51.26 ± 8.23	≤0.001

**Table 3:** Comparison of elastic modulus at different stress levels.

Stress (MPa)	Vertical (MPa)	Horizontal (MPa)	*P*
0.01	1.28 ± 0.70	1.17 ± 0.50	0.338
0.02	1.57 ± 0.67	1.46 ± 0.47	0.373
0.03	1.84 ± 0.64	1.75 ± 0.45	0.417

## Data Availability

The data that support the findings of this study are available from the corresponding author upon reasonable request.
